# Wireless Localized Electrical Stimulation Generated by an Ultrasound‐Driven Piezoelectric Discharge Regulates Proinflammatory Macrophage Polarization

**DOI:** 10.1002/advs.202100962

**Published:** 2021-05-03

**Authors:** Ying Kong, Feng Liu, Baojin Ma, Jiazhi Duan, Wenhu Yuan, Yuanhua Sang, Lin Han, Shuhua Wang, Hong Liu

**Affiliations:** ^1^ State Key Laboratory of Crystal Materials Shandong University Jinan Shandong 250100 China; ^2^ Department of Periodontology School and Hospital of Stomatology Cheeloo College of Medicine Shandong University & Shandong Key Laboratory of Oral Tissue Regeneration & Shandong Engineering Laboratory for Dental Materials and Oral Tissue Regeneration Jinan Shandong 250012 China; ^3^ Jinan Biobase Biotech Co., Ltd Jinan 250100 China; ^4^ Institue of Marine Science and Technology Shandong University Qingdao Shandong 266200 China; ^5^ Advanced Medical Research Institute Shandong University Jinan Shandong 250100 China; ^6^ Institute for Advanced Interdisciplinary Research University of Jinan Jinan Shandong 250022 China

**Keywords:** electrical stimulation, macrophage polarization, piezoelectric materials, proinflammatory, ultrasound

## Abstract

Proinflammatory (M1) macrophages play a vital role in antitumor immunity, and regulation of proinflammatory macrophage polarization is critical for immunotherapy. The polarization of macrophages can be regulated by biological or chemical stimulation, but investigations of the regulatory effect of physical stimulation are limited. Herein, regulating macrophage polarization with localized electrical signals derived from a piezoelectric *β*‐phase poly(vinylidene fluoride) (*β*‐PVDF) film in a wireless mode is proposed. Charges released on the surface of the *β*‐PVDF film driven by ultrasonic irradiation can significantly enhance the M1 polarization of macrophages. Mechanistic investigation confirms that electrical potentials rather than reactive oxygen species and mechanical forces enable Ca^2+^ influx through voltage‐gated channels and establishment of the Ca^2+^‐CAMK2A‐NF‐*κ*B axis to promote the proinflammatory macrophage response during ultrasound treatment. Piezoelectric material‐mediated electrical signal‐activated proinflammatory macrophages significantly inhibit tumor cell proliferation. A method for electrogenetic regulation of immune cells as well as a powerful tool for engineering macrophages for immunotherapy is provided here.

## Graphical abstract

Regulating macrophage polarization with localized electrical signals derived from a piezoelectric *β*‐phase poly(vinylidene fluoride) (*β*‐PVDF) film in a wireless mode is proposed, providing a method for electrogenetic regulation of immune cells as well as a powerful tool for engineering macrophages for immunotherapy.

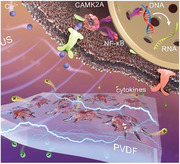

## 1 Introduction

Innate immune cells play a critical role in many biological processes and disease therapies, especially tumor immunotherapy.^[^ ^1^, ^2^, ^3^ ^]^ Macrophages are one of the most important innate immune cells and have different polarization states,^[^ ^4^, ^5^ ^]^ including the proinflammatory (M1) phenotype and anti‐inflammatory (M2) phenotype. Proinflammatory macrophages kill cancer cells or inhibit tumor growth, and regulating the M1 polarization of macrophages has become a very hot topic in the cell technology field.^[^ ^6^ ^]^ The immune process and macrophage polarization are routinely regulated by biological and chemical factors. ^[^ ^7^ ^]^ For example, immune homeostasis is substantially altered due to inflammation stimulated by microbial danger signals, which are defined as pathogen‐associated molecular patterns (PAMPs) that involve microorganisms, lipopolysaccharide (LPS), DNA, RNA, etc.^[^ ^8^ ^]^ Macrophages not only respond to PAMPs through pattern recognition receptors (PRRs)^[^ ^9^ ^]^ but also initiate the immune response through various microenvironment receptors, which sense temperature,^[^ ^10^ ^]^ pH,^[^ ^11^ ^]^ osmotic pressure,^[^ ^12^ ^]^ oxygen,^[^ ^13^ ^]^ etc. Although much progress has been made in elucidating the mechanism underlying the regulation of the immune cell response based on PRRs and microenvironmental sensors, it is still a great challenge to identify rapid and highly efficient methods that drive the M1 polarization of macrophages.^[^ ^6^ ^]^

Recently, there has been great interest in the regulation of immune cells by physical signals.^[^ ^14^, ^15^, ^16^ ^]^ As an important type of physical stimulation, electrical signals have aroused increasing attention in the biomedical field and can directly program cellular behavior in an electrogenetic‐mediated manner.^[^ ^17^, ^18^ ^]^ However, few studies have focused on how electrical signals regulate macrophage polarization.^[^ ^19^, ^20^ ^]^ Under physiological or pathological conditions, cell death and tissue damage cause ion current fluctuations in the cellular microenvironment,^[^ ^21^ ^]^ which also affect the electrical potential of the microenvironment. Therefore, electrical stimulation may have an important effect on macrophage polarization through regulating the cellular microenvironment.

Cell fate decisions can be directed through voltage‐sensitive receptors under electrical stimulation. When macrophages are seeded on the surface of a conductive material and electrical stimulation is applied via connected wires and electrodes, the macrophages cannot sense local electrical stimulation due to the general distribution of the electric potential on surface of the material. *β*‐Phase polyvinylidene fluoride (*β*‐PVDF) has an all‐trans conformation and exhibits a net dipole moment,^[^ ^22^ ^]^ which has a perpendicular polarization direction and is recognized as a superior piezoelectric material. The electroactive *β*‐PVDF structures have shown great potential in various applications, such as the sensors and actuators, energy generation and storage, tissue engineering, and biomedicine. ^[^ ^23^, ^24^ ^]^ Piezoelectric effect can also tailor bacteria response by regulating electrical microenvironments under mechanical stimulation.^[^ ^25^ ^]^ Inspired by these investigations, the charges released on the surface of the *β*‐PVDF film can be driven by ultrasonic irradiation due to the deformation of the film, and these localized electrical signals can be sensed by voltage‐sensitive receptors on macrophages, thus enhancing or regulating their polarization.

Here, we designed a strategy to noninvasively enhance the proinflammatory response of macrophages through piezoelectric material‐meditated localized electrical signals with ultrasound assistance. This strategy relies on the microvibration of the *β*‐PVDF film under ultrasound treatment and the release of a localized charge via the spontaneous polarization of the crystalline phase, which significantly triggers the selective expression and secretion of proinflammatory chemoattractant factors. Based on RNA sequencing, Ca^2+^ influx through voltage‐gated channels is promoted, and a Ca^2+^‐CAMK2A‐NF‐*κ*B axis is established to induce M1 macrophage polarization. Following coculture with M1‐polarized macrophages, tumor cell viability is significantly inhibited due to macrophage‐secreted inflammatory factors, suggesting the potential applications of this strategy as a tumor immunotherapy. Our study strongly supports that piezoelectric material‐mediated electrical potentials mainly regulate immunological responses, providing controllable noninvasive stimulus to drive the inflammatory response.

## 2 Results and Discussion

### 2.1 Material Characterization

**Figure 1:**
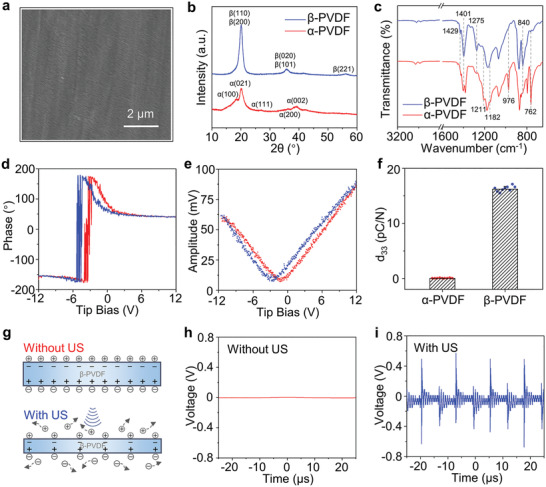
Material characterization. a) SEM image of *β*‐PVDF. b) XRD patterns of *α*‐PVDF and *β*‐PVDF. c) FT‐IR spectra of *α*‐PVDF and *β*‐PVDF. d) Phase curve and e) amplitude curve of *β*‐PVDF at ramp voltages from −12 to 12 V. f) The d_33_ constant of *α*‐PVDF and *β*‐PVDF. g) Illustration of the ultrasound‐triggered piezoelectric effect. The output voltage of *β*‐PVDF h) without or i) with US. US, ultrasound stimulation.

The surface morphologies of the *β*‐PVDF and *α*‐PVDF films were observed by scanning electron microscopy (SEM). As shown in **Figure** **1a** and Figure S1 in the Supporting Information, the *β*‐PVDF and *α*‐PVDF films exhibit a flat and smooth morphology. The X‐ray diffraction (XRD) patterns of *β*‐phase PVDF and *α*‐phase PVDF can be easily indexed (Figure 1b) and correspond to JCPDS cards #42‐1649 and #42‐1650, respectively. As shown in Figure 1c, the characteristic absorption peaks of *β*‐PVDF in the Fourier transforming‐infrared spectrum (FT‐IR) spectra can be observed at 1429, 1401, 1275, and 840 cm^–1^. The peak at 1429 cm^–1^ are assigned to CH_2_ scissoring vibration, and CH_2_ wagging and CC asymmetrical stretching vibration collectively contribute to the peak at 1401 cm^–1^. The peak at 1275 cm^–1^ is related to CF_2_ symmetrical stretching, CC symmetrical stretching and CCC scissoring vibration, and that at 840 cm^–1^ is assigned to CH_2_ out‐of‐plane bending and CF_2_ asymmetrical stretching vibration.^[^ ^26^ ^]^ In addition, the characteristic peaks at 1211 cm^–1^ (CF_2_ asymmetrical stretching and CH_2_ wagging vibration), 1182 cm^–1^ (CF_2_ symmetrical stretching and CH_2_ twisting vibration), 976 cm^–1^ (CH_2_ twisting vibration) and 762 cm^–1^ (CF_2_ scissoring and CCC scissoring vibration) are predominantly indicative of *α*‐phase PVDF.^[^ ^26^ ^]^

It is generally known that poled *β*‐phase PVDF has superior piezoelectric performance among various polymers. To evaluate the piezoelectric response, the properties of *β*‐PVDF were evaluated via piezoelectric force microscopy (PFM) with a conductive probe. Piezoelectric hysteresis and butterfly loops are observed at a tip bias between −12 and 12 V (Figure 1d,e). The rhomboid phase hysteresis loop and “butterfly shape” amplitude loop are indicative of the representative piezoelectric response and polarization switching behavior of the *β*‐PVDF film. No phase hysteresis loop or amplitude butterfly curve can be observed for the *α*‐PVDF sample (Figure S2, Supporting Information). The piezoelectric constants d_33_ of *α*‐PVDF and *β*‐PVDF are 0.02 and 16.22 pC N^−1^, respectively (Figure 1f), calculated as the average of ten sites on two samples.

*β*‐PVDF film exhibits electroneutrality without ultrasound stimulation (US), because the value of net charges absorbed on the surface is equal to that of internal charges; however, since piezoelectric materials deform and generate an internal electric field under mechanical force, *β*‐PVDF can produce electric signals and accelerate charge release from its both sides with ultrasound stimulation (Figure 1g). Therefore, the surface charge variation can be sensed by cells with ultrasound stimulation and thus affects cell behavior. To detect the dynamic change in charge release, the two sides of *β*‐PVDF were covered with copper foil and connected with wires (Figure S3, Supporting Information). In the absence of US, the output voltage from both sides is close to 0 (Figure 1h), as detected by an electronic oscilloscope. As Figure 1i shows, the output voltage can reach tens to hundreds of millivolts under US with a frequency of 80 kHz ultrasound. The voltage fluctuation period is ≈12.5 µs, which is in accord with the US frequency. The charge release and output voltage of *β*‐PVDF laid the foundation for noninvasive and localized electric stimulation of attached cells. We have also taken the effect of culture media on the surface charge variation into consideration. The composition of cell culture medium is complex, consisting of various amino acids, vitamins, carbohydrates, and inorganic ions. In the following cell experiments, all groups were cultured in the same cell culture medium and kept consistent in other conditions. Cells have a direct contact with materials, and the released charges can be immediately sensed by cell membranes; therefore, the effect of surface charges on culture medium is negligible with such a short ultrasound treatment.

It is worth mentioning that a gold layer was deposited on *β*‐PVDF film in the cell experiments. The contact angle changes from ≈82.70° to ≈78.25° after gold layer deposition, which indicates that gold layer modifies the contact angle and slightly improves the surface wettability (Figure S4, Supporting Information). The piezoelectric response maintained when *β*‐PVDF film was coated with the gold layer, as shown in Figure S5 in the Supporting Information. More importantly, the gold layer on *β*‐PVDF film is beneficial to accumulate the generated charges during ultrasonic treatment, which can be sensed by adhesive cells.

### 2.2 Cytocompatibility of *β*‐PVDF

**Figure 2:**
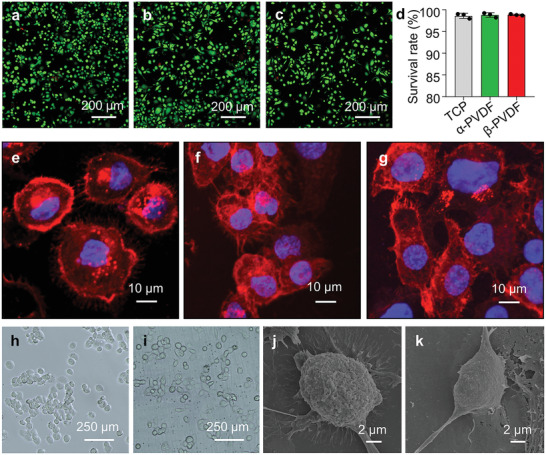
Cytocompatibility of *β*‐PVDF. Merged images of live (green) and dead (red) PMA‐pretreated THP‐1 cells seeded on a) TCP, b) *α*‐PVDF, and c) *β*‐PVDF for 24 h. d) The survival rate of PMA‐pretreated THP‐1 cells on TCP, *α*‐PVDF, and *β*‐PVDF (mean ± SD, *n* = 3). Images of F‐actin (red) and nuclear (blue) staining of cells on e) TCP, f) *α*‐PVDF, and g) *β*‐PVDF for 24 h. Bright field images of PMA‐pretreated THP‐1 cells on h) TCP and i) *β*‐PVDF for 24 h. Representative spreading morphology of THP‐1 cells on j) TCP and k) *β*‐PVDF for 24 h. The experiments were performed three times independently.

Phorbol‐12‐myristate 13‐acetate (PMA) can induce the adherence of THP‐1 cells, thereby transforming monocytes into M0 macrophages. To investigate cytocompatibility, THP‐1 cells were pretreated with PMA on tissue culture plates (TCPs), *α*‐PVDF and *β*‐PVDF, and then live/dead staining was used to stain live cells green with calcein acetoxymethyl ester (calcein AM) and stain dead cells red with propidium iodide (PI) after 24 h of cultivation (**Figure** **2a**–c). The overwhelming majority of cells were live, and a small minority of cells were dead. Figure S6 in the Supporting Information shows individual images of live and dead cells on different substrates. The statistical survival rates of macrophages cultured on TCP, *α*‐PVDF, and *β*‐PVDF were 99.00 ± 0.26%, 98.64 ± 0.60%, and 98.65 ± 0.38%, respectively (Figure 2d). The high survival rate of PMA‐pretreated THP‐1 cells indicates that *α*‐PVDF and *β*‐PVDF display good cytocompatibility. The results of the Cell Counting Kit‐8 (CCK‐8) assay also suggested that *β*‐PVDF has good biocompatibility (Figure S7, Supporting Information). As a positive control, doxorubicin (DOX) was used to induce cell death and representative live/dead staining images show similar morphology of live cells and dead cells to our study (Figure S8, Supporting Information). Cells also keep good viability after cultured on *β*‐PVDF film for 72 h (Figure S9, Supporting Information).

The adhesive structure of PMA‐pretreated THP‐1 cells can be altered by different substrates. Therefore, we stained cells with F‐actin to observe the adherent cells on TCP, *α*‐PVDF, and *β*‐PVDF at 24 h (Figure 2e–g). The PMA‐pretreated THP‐1 cells in the *α*‐PVDF group (Figure 2f) and *β*‐PVDF group (Figure 2g) exhibited a more elongated cell shape than those in the TCP group (Figure 2e) which exhibited a round cell shape; this finding may prove that macrophages on PVDF substrates are polarization. F‐actin and nuclear staining images of cells on different substrates are shown in Figure S10 in the Supporting Information. The F‐actin staining images under lower magnification indicate that *β*‐PVDF group favors cell elongation than TCP group (Figure S11a,b, Supporting Information). The cell aspect ratio of *β*‐PVDF group is significantly higher than that of TCP group (Figure S11c, Supporting Information), suggesting that the shape of macrophages on *β*‐PVDF substrate is elongated.

Bright field images of PMA‐pretreated THP‐1 cells on TCP and *β*‐PVDF are shown in Figure 2h,i, respectively. Based on the representative SEM images of PMA‐pretreated THP‐1 cells on TCP and *β*‐PVDF (Figure 2j,k), *β*‐PVDF affects cellular morphology and tends to drive macrophage polarization more robustly than TCP. The good cytocompatibility of *β*‐PVDF and elongated morphology of the cells indicate that the *β*‐PVDF film is appropriate for cell culture and macrophage regulation.

### 2.3 The Piezoelectric Effect Enhances the M1 Polarization of Macrophages with Ultrasound Irradiation

**Figure 3:**
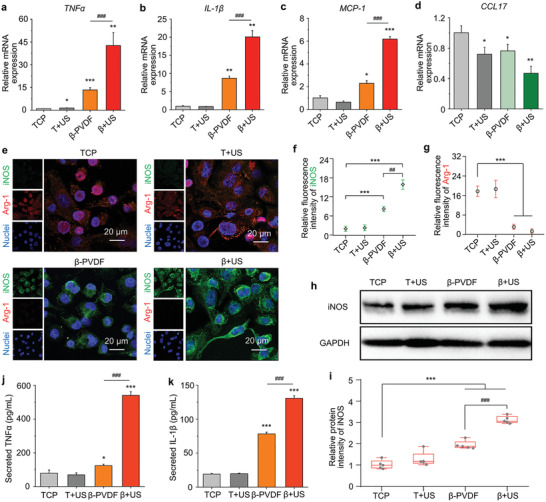
The piezoelectric effect enhances the M1 polarization of macrophages with ultrasound irradiation. Relative mRNA expression of the M1 markers a) TNF‐*α*, b) IL‐1*β*, and c) MCP‐1 and d) the M2 marker CCL17 in macrophages cultured on TCP, T+US, *β*‐PVDF, and *β*+US for 3 d (T, TCP; *β*, *β*‐PVDF; US, ultrasound stimulation). The data are presented as the mean ± SD and were normalized to the level in the TCP group. e) Immunofluorescence staining images of iNOS (an M1 marker) and Arg‐1 (an M2 marker) after 3 d of culture. Statistical analysis of f) iNOS and g) Arg‐1 fluorescence intensity by ImageJ software. h) Western blot analysis of iNOS protein expression in cells on TCP, T+US, *β*‐PVDF, and *β*+US. i) Statistical analysis of iNOS protein intensity in (h) by ImageJ software. j) Secreted TNF‐*α* level and k) secreted IL‐1*β* level on day 3, as measured by ELISA. Significance was determined by unpaired two‐tailed Student's *t*‐test (^*^ *p* < 0.05, ^**^ *p* < 0.01, ^***^ *p* < 0.001 vs the TCP control group, ^###^ *p* < 0.001 vs the *β*‐PVDF group (*n* = 3 exception for (i) (*n* = 5)). The experiments were performed three times independently.

According to previous reports, physical cues (mechanical force^[^ ^15^ ^]^ and magnetism,^[^ ^16^ ^]^ etc.) play important roles in the fate decision of immune cells; therefore, we propose that these cues meditated by nanomaterials have a similar effect on macrophages. To investigate the effect of material‐meditated physical cues on macrophage polarization, we applied US to cells cultured in the piezoelectric *β*‐PVDF phase for noninvasive and noncontact stimulation. Since tumor necrosis factor‐*α* (TNF‐*α*), interleukin‐1*β* (IL‐1*β*), and monocyte chemotactic protein 1 (MCP‐1) are typical markers of M1 macrophages,^[^ ^4^ ^]^ we evaluated the expression of these genes by real‐time quantitative polymerase chain reaction (RT‐qPCR). Macrophages from the *β*‐PVDF group not exposed to US showed higher mRNA expression of M1 markers than macrophages from the TCP group, as shown in **Figure** **3a**–c, indicating that *β*‐PVDF itself promotes the M1 polarization of macrophages; this finding is consistent with a previous report.^[^ ^27^ ^]^ Interestingly, following US treatment on *β*‐PVDF (termed as *β*+US), significant upregulation of the mRNA levels of M1 markers compared with those in the TCP (≈42.7‐fold, 20.1‐fold, and 6.2‐fold for TNF‐*α*, IL‐1*β*, and MCP‐1, respectively) and *β*‐PVDF groups (≈3.2‐fold, 2.3‐fold, and 2.7‐fold for TNF‐*α*, IL‐1*β*, and MCP‐1, respectively) was observed (Figure 3a–c), proving that the piezoelectric effect of *β*‐PVDF greatly enhances M1 polarization. Besides, we also examined the macrophage polarization at early time. The mRNA levels of M1 markers were also enhanced in *β*+US group compared with those in the TCP and *β*‐PVDF groups at 1 d (Figure S12, Supporting Information). However, US treatment on TCP (termed T+US) had almost no impact on the M1 polarization of macrophages. The mRNA expression of the M2 marker C–C motif chemokine ligand 17 (CCL17) (Figure 3d) was slightly inhibited in the *β*+US group compared with the TCP group. Therefore, we propose that the piezoelectric effect rather than ultrasound acts as a critical factor in M1 polarization and that it also inhibits the M2 polarization of macrophages.

Next, we stained for the intracellular M1 marker inducible nitric oxide synthase (iNOS) and M2 marker Arginase‐1 (Arg‐1) to observe the extent of macrophage polarization (Figure 3e). More Arg‐1 staining than iNOS staining was found in the TCP and T+US groups. In contrast, almost all the macrophages in the *β*+US group showed positive staining for iNOS, and more macrophages in this group than in the TCP, T+US, and *β*‐PVDF groups exhibited staining. Statistical analysis of the replicated experiments indicated that the iNOS fluorescence intensity (Figure 3f) was higher in the *β*+US group than in the control groups, while the Arg‐1 fluorescence intensity (Figure 3g) in the *β*+US group was the lowest among all groups. As revealed by western blot analysis, the expression of iNOS in the *β*+US group was the highest among all the groups (Figure 3h,i). These results verify that macrophage M1 polarization is enhanced at the intracellular protein level by the piezoelectric effect with ultrasound irradiation.

To further evaluate the extent of M1 polarization, the secretion of related inflammatory factors was quantified by enzyme‐linked immunosorbent assay (ELISA). The levels of TNF‐*α* and IL‐1*β* secreted from macrophages from the *β*+US group were significantly higher than those secreted from macrophages from the other groups, suggesting a more robust inflammatory response (Figure 3j,k). Taken together, these data support the viewpoint that the piezoelectric effect enhances the M1 polarization of macrophages and inhibits M2 polarization.

### 2.4 Mechanism of Piezoelectrically Enhanced M1 Macrophage Polarization

Mechanical force^[^ ^15^ ^]^ and ROS^[^ ^28^ ^]^ exert regulatory effects on macrophages by changing their phenotypes, while electrical potential may also play a critical role in macrophage fate decisions. These three signals can be simultaneously generated by US treatment; thus, it is necessary to distinguish their roles and mechanisms in regulating the macrophage inflammatory response on piezoelectric substrates. Therefore, comprehensive analysis of gene expression profiles in the TCP, T+US, *β*‐PVDF, and *β*+US groups was performed by RNA sequencing to investigate the underlying mechanism of piezoelectric effect‐induced polarization.

**Figure 4:**
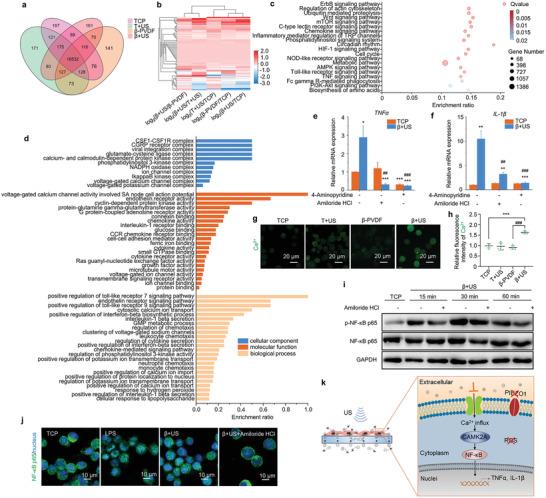
Polarization mechanism of the piezoelectric effect. a) Venn diagram of differentially expressed genes between the TCP, T+US, *β*‐PVDF, and *β*+US groups. b) Heat map of differentially clustered voltage‐gated ion channels. c) KEGG pathway enrichment analysis of differentially expressed genes between the TCP and *β*+US groups. The 20 most significantly enriched pathways are shown. d) GO classification of differentially expressed genes between the TCP and *β*+US groups related to the cellular component, molecular function, and biological process categories (*p* < 0.05 for cellular component, *p* < 0.01 for molecular function and biological process). e) TNF‐*α* and f) IL‐1*β* mRNA expression in macrophages in the TCP and *β*+US group following treatment with 4‐aminopyridine and amiloride HCl. Significance was determined by unpaired two‐tailed Student's *t*‐test (^**^ *p* < 0.01, ^***^ *p* < 0.001 vs the *β*+US group (*n* = 3)). g) Detection of Ca^2+^ influx in macrophages in the TCP, T+US, *β*‐PVDF and *β*+US groups. h) Statistical analysis of Ca^2+^ fluorescence intensity by ImageJ software. Significance was determined by unpaired two‐tailed Student's *t*‐test (^***^ *p* < 0.001 vs the TCP control group, ^###^ *p* < 0.001 vs the *β*‐PVDF group (*n* = 5)). i) Western blot analysis of NF‐*κ*B p65 protein expression in the TCP and *β*+US groups. j) Immunofluorescence staining for NF‐*κ*B p65 in the TCP, *β*+US and *β*+US+amiloride HCl groups. k) Schematic illustration of the polarization mechanism of the piezoelectric effect. The experiments were performed three times independently.

As shown in **Figure** **4a**, 16532 genes were expressed in all four groups, and the number of genes exclusively expressed in each group was 167 (TCP), 171 (T+US), 141 (*β*‐PVDF), and 161 (*β*+US). Moreover, the numbers of differentially expressed genes in the *β*+US group were 438 (in comparison with the TCP group), 410 (in comparison with the T+US group), and 485 (in comparison with the *β*‐PVDF group). Voltage‐sensitive channels, which are expressed in a variety of cell types, can mediate the entry of ions into excitable cells, which can be affected by the membrane potential, and these channels are also involved in a variety of cellular physiological processes.^[^ ^29^, ^30^ ^]^ To investigate the expression of genes associated with voltage, including sodium voltage‐gated channels, potassium voltage‐gated channels, calcium voltage‐gated channels, voltage‐dependent anion channels, chloride voltage‐gated channels, and transient receptor potential cation channels, we generated a heat map showing differential clustering of related genes (Figure 4b) and heat maps showing differential clustering of genes associated with six different kinds of voltage‐gated ion channels (Figure S13, Supporting Information). The color scale indicates that several important genes, such as SCN8A, KCNH2, KCNB1, KCNC1, KCNAB1, CACNA1C, CNCNB1, CACNA2D1, CACNA1H, CNCNA1A, and CACNA1G, are significantly upregulated on *β*‐PVDF with ultrasound treatment compared to under the other conditions. Among these genes, the voltage‐gated sodium channel SCN8A is associated with macrophage migration;^[^ ^31^ ^]^ the potassium voltage‐gated channels KCNH2,^[^ ^32^ ^]^ and KCNC1^[^ ^33^ ^]^ are involved in the regulation of inflammatory factor secretion and lymphocyte activation; and voltage‐gated calcium channels, including CACNA1C,^[^ ^34^, ^35^ ^]^ CACNA1H,^[^ ^36^, ^37^ ^]^ CNCNA1A,^[^ ^38^ ^]^ and CACNA1G,^[^ ^39^ ^]^ can enhance the inflammatory effect of macrophages and contribute to shaping T helper cell cytokine profiles. Therefore, the results indicate that these upregulated voltage‐gated channels may play an important role in the piezoelectric effect‐induced promotion of the macrophage polarization to the inflammatory phenotype.

Kyoto Encyclopedia of Genes and Genomes (KEGG) analysis was used to identify signaling pathways that were significantly enriched in differentially expressed genes between the TCP and *β*+US groups in detail (Figure 4c). Among these pathways, the ErbB signaling pathway, mTOR signaling pathway, HIF‐1 signaling pathway, TNF signaling pathway, and Toll‐like receptor signaling pathway were strongly associated with the M1 polarization of macrophages. According to KEGG analysis, piezoelectric material‐mediated electrical stimulation activated the ErbB signaling pathway (Figure S14, Supporting Information), which can enhance the inflammatory response of macrophages.^[^ ^40^ ^]^ Activation of metabolic sensors in the mTORC1‐Akt axis supports macrophage activation.^[^ ^41^ ^]^ Notably, TCS‐mTORC1 in the mTOR signaling pathway (Figure S15, Supporting Information) was significantly activated in the *β*+US group compared to the TCP group, indicating that piezoelectric material‐mediated electrical stimulation can enhance the macrophage inflammatory response through activation of the mTOR signaling pathway. In addition, HIF‐1*α* was significantly upregulated by piezoelectric material‐mediated electrical stimulation in the *β*+US group (Figure S16, Supporting Information), suggesting that HIF‐1*α* may be important for regulating macrophage inflammatory activation after ultrasound‐induced piezoelectric treatment, contributing to the inflammatory response of macrophages by binding to the promoter of IL‐1*β*.^[^ ^42^ ^]^

To explore the functions of polarized macrophages, gene ontology (GO) functional enrichment analysis of differentially expressed genes between the TCP and *β*+US groups related to the cellular component, molecular function, and biological process categories was performed, and the results are shown in Figure 4d. Many of the significantly affected GO terms in the cellular component category were related to voltage‐gated ion channel complexes, such as voltage‐gated calcium channel complexes and voltage‐gated potassium channel complexes; calcium‐ and calmodulin‐dependent protein kinase complexes were also enriched. Moreover, typical complexes closely associated with macrophage polarization were enriched, including the CSF1‐CSF1R complex, CGRP receptor complex, glutamate‐cysteine ligase complex, IkappaB kinase complex, etc. For the molecular function category, several GO terms associated with macrophage function, including endothelin receptor activity, chemokine activity, interleukin‐1 receptor binding, CCR chemokine receptor binding, etc., were significantly enriched in genes expressed in the *β*+US group. Voltage‐gated ion channel activity was also a significantly enriched term in the molecular function category. Furthermore, the significantly enriched biological process terms were closely associated with macrophage polarization, such as the endothelin receptor signaling pathway, positive regulation of interleukin‐1*β* secretion, regulation of phosphatidylinositol 3‐kinase activity, and the chemokine‐mediated signaling pathway. Some biological processes related to voltage‐gated ion channels, such as clustering of voltage‐gated sodium channels, positive regulation of potassium ion transmembrane transport, and positive regulation of calcium ion import, were also significantly enriched. Therefore, based on the identified GO terms, piezoelectric material‐mediated electric potential activates voltage‐gated ion channels to effectively enhance M1 macrophage polarization.

To further determine the role of voltage‐sensitive channels, typical inhibitors (the T‐type calcium channel blocker amiloride HCl and the K^+^ channel blocker 4‐aminopyridine) were used to pretreat THP‐1 cells for 1 h. After US treatment for 3 d, the gene expression of inflammatory factors in macrophages was investigated by qPCR analysis. The amiloride HCl treatment group and 4‐AP treatment group both showed significantly lower mRNA expression of TNF‐*α* and IL‐1*β* than that of no inhibitor US treatment group, while these inhibitors had no significant effect on the TCP group (Figure 4e,f), meaning that voltage‐gated ion channels may participate in piezoelectricity‐mediated polarization of macrophages.

To verify whether the piezoelectric effect can specifically activate ion channels and increase the intracellular Ca^2+^ concentration and signal transduction, Fluo‐4 AM staining was performed to detect the Ca^2+^ influx in THP‐1 cells on *β*‐PVDF or TCP with US treatment after 20 min. Quantification of fluorescence intensity indicated that the Ca^2+^ concentration in THP‐1 cells in the *β*+US group was significantly higher than that in THP‐1 cells in the other groups under the same intensity of light excitation at 488 mm (Figure 4g,h). In addition, based on analysis of calcium/calmodulin kinase family expression, calcium‐regulated CAMK2A gene expression was significantly upregulated in the *β*+US group compared to the other groups (Figure S17, Supporting Information), which is in accordance with the significantly enriched calcium‐ and calmodulin‐dependent protein kinase complex GO terms. According to a previous report, the NF‐*κ*B signaling pathway plays an important role in macrophage polarization, and its activation is highly correlated with calcium ions^[^ ^43^ ^]^ and CAMK2A levels.^[^ ^44^ ^]^ Western blot analysis revealed that piezoelectric material‐mediated electrical stimulation promoted NF‐*κ*B p65 phosphorylation, while amiloride HCl significantly inhibited this process (Figure 4i). Immunofluorescence staining showed that amiloride HCl inhibited the nuclear translocation of NF‐*κ*B p65 induced by piezoelectric material‐mediated electrical stimulation (Figure 4j), further confirming that the ability of piezoelectric material‐mediated electric signals to induce the inflammatory response of macrophages is dependent on the electrical potential‐Ca^2+^‐CAMK2A‐NF‐*κ*B signaling circuit.

In addition to those of electric potential, the roles of mechanical force and ROS in the polarization should be identified. To distinguish the effects of mechanical force from those of other cues, THP‐1 cells were cultured on nonpiezoelectric *α*‐PVDF with US treatment for 3 d to analyze the gene expression of related inflammatory factors. The results showed that US had no obvious effect on TNF‐*α* and IL‐1*β* mRNA expression in cells on *α*‐PVDF (Figure S18, Supporting Information). Cyclical hydrostatic pressure, a kind of mechanical force, can initiate the macrophage inflammatory response via the mechanically activated ion channel PIEZO1.^[^ ^15^ ^]^ We also detected the gene expression of PIEZO1 in cells cultured on TCP, T+US, *β*‐PVDF, and *β*+US and found that the mRNA expression level was not altered by mechanical force during the response to piezoelectric signals (Figure S19a, Supporting Information). The heat maps showing differential clustering of PIEZO1 and PIEZO2 based on RNA‐sequencing data are consistent with the RT‐qPCR results (Figure S19b, Supporting Information). Therefore, mechanical force does not play a key role in M1 macrophage polarization during ultrasound treatment.

ROS are additional important factors that affect macrophage polarization;^[^ ^28^ ^]^ thus, we next evaluated the function of ROS in altering the inflammatory macrophage phenotype during the US‐triggered piezoelectric response. Dichlorodihydrofluorescein diacetate (DCFH‐DA) staining was carried out to verify whether material‐mediated piezoelectric stimulation can elevate intracellular ROS levels. As shown in Figure S20 in the Supporting Information, there was no significant increase in intracellular ROS levels in the *β*+US group compared to other groups. Additionally, the TNF‐*α* and IL‐1*β* mRNA levels in the glutathione (GSH)‐treated *β*+US group, in which ROS were scavenged, were similar to those in the untreated *β*+US group, indicating that ROS are not crucial factors (Figure S21, Supporting Information).

Taken together, these results suggest that enhanced M1 polarization in the *β*+US group is driven by electrical potential during US treatment, while mechanical forces and ROS do not exert effects. The electrical potential enables Ca^2+^ influx through voltage‐gated calcium channels and upregulation of CAMK2A, thus promoting the nuclear translocation of NF‐*κ*B and inducing the expression of inflammatory factors (Figure 4k).

### 2.5 Enhancement of M1 Macrophage Polarization by the Piezoelectric Effect Inhibits Tumor Cell Growth

**Figure 5:**
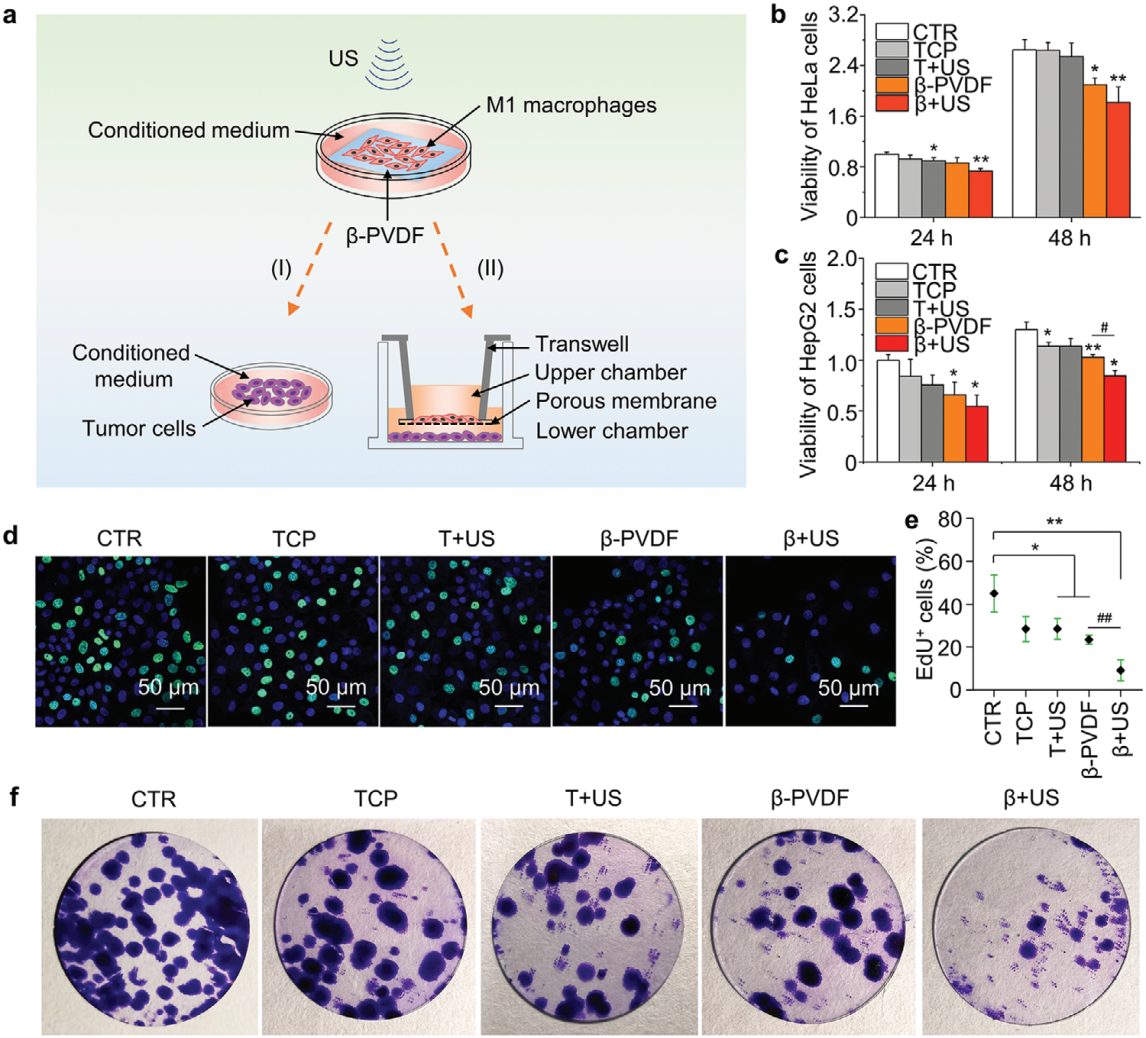
Coculture of macrophages and tumor cells. a) Illustration of the cocultures. Viability of b) HeLa cells and c) HepG2 cells cultured with medium conditioned by macrophages in the TCP, T+US, *β*‐PVDF, and *β*+US groups after 3 d of culture normalized to the viability of the untreated control group (CTR). d) Analysis of the proliferation of HepG2 cells in the CTR monoculture group and those cocultured with macrophages in the TCP, T+US, *β*‐PVDF, and *β*+US for 3 d by the EdU assay. EdU is indicated by green fluorescence, while nuclei are blue. e) Statistical analysis of EdU^+^ HepG2 cells by ImageJ software. f) Colony formation by HepG2 cells cultured in 24‐well tissue culture plates in the CTR monocultured group and those cocultured with macrophages in the TCP, T+US, *β*‐PVDF, and *β*+US groups for 10 d. Significance was determined by unpaired two‐tailed Student's *t*‐test (^*^ *p* < 0.05, ^**^ *p* < 0.01 vs the CTR control group, ^#^ *p* < 0.05, ^##^ *p* < 0.01 vs the *β*‐PVDF group (*n* = 3)). The experiments were performed three times independently.

M1 macrophages release inflammatory factors, such as TNF‐*α*, IL‐1*β*, and interleukin‐6 (IL‐6), which are believed to exert cytocidal effects against tumor cells. Here, we next sought to investigate whether polarized macrophages can inhibit the viability and proliferation of tumor cells. Two strategies were designed to evaluate the function of polarized macrophages (**Figure** **5a**): I) Conditioned medium containing cytokines released from macrophages were used to stimulate tumor cells. II) To evaluate their interaction, macrophages and tumor cells were seeded in the upper chamber and the lower chamber of a Transwell coculture system, respectively.

M1 macrophage‐conditioned medium contains various kinds of inflammatory factors that may inhibit tumor cell viability. After macrophages in TCP, T+US, *β*‐PVDF, and *β*+US groups were polarized for 3 d, we collected macrophage‐conditioned medium to stimulate HeLa cells and HepG2 cells. The negative control group not treated with conditioned medium was termed the CTR group. The results of the CCK‐8 assay suggested that the viability of both HeLa cells (Figure 5b) and HepG2 cells (Figure 5c) treated with macrophage‐conditioned medium collected from the *β*+US group after 3 d of culture was lower than that of HeLa and HepG2 cells from the other groups, indicating that more antitumor cytokines were secreted in medium from the *β*+US group.

In the Transwell coculture system, merging of the green signal (EdU) with the blue signal (nuclei) indicated that macrophages in the *β*+US group more obviously inhibited the proliferation of HepG2 cells than macrophages in the CTR monoculture group and the other coculture groups after 3 d (Figure 5d,e). We also assessed the effect of macrophages on HepG2 cells for 10 d through a colony formation experiment, as shown in Figure 5f. Compared with the CTR, macrophages in the *β*+US group significantly inhibited the formation of HepG2 cell colonies, and *β*+US group macrophages showed the lowest colony forming efficiency among all groups. Similar antitumor effects were observed when M1 macrophages and HeLa cells were cocultured, as indicated by the EdU assay (Figure S22, Supporting Information). Therefore, M1 macrophages in the *β*+US group suppress the viability and proliferation of tumor cells, and piezoelectric material‐mediated electrical signals may have potential use for local tumor immunotherapy, such as skin cancer treatment.

## 3 Conclusion

In this study, we demonstrated that localized electrical charge release driven by ultrasonic irradiation on a *β*‐PVDF film significantly enhanced the M1 polarization of macrophages. The gene expression of M1 markers on *β*‐PVDF film with ultrasound stimulation was several to dozens of times that in the control group, and the same trend was observed for intracellular and secreted protein. The enhancement of M1 polarization and simultaneous inhibition of M2 polarization localized electrical signals were primarily due to Ca^2+^ influx through voltage‐gated channels and the Ca^2+^‐CAMK2A‐NF‐*κ*B axis, which promote the release of the proinflammatory factors TNF‐*α* and IL‐1*β*. Proinflammatory macrophages exerted cytocidal effects against tumor cells in both the conditioned medium and coculture experiments. This wireless electrical stimulation, which is cheap, highly efficient, and easy to use, allows regulation of macrophage fate. Our study reveals that piezoelectric material‐meditated electrical signals can controllably drive the inflammatory response with noninvasive ultrasound assistance and proposes an electrogenetic method for regulating the fate of immune cells and providing tumor immunotherapy. There are mainly three aspects that can be furtherly explored according to our findings: 1) adoptive cellular therapy—culturing macrophages of patients on piezoelectric materials and applying ultrasound stimulation in vitro, then polarized macrophages will be harvested and infiltrated at the tumor site of the patients by intravenous injection, or reach the solid tumor by intratumor injection for immunotherapy; 2) postoperative therapy—causing effective damage to residual tumor cells and bacterial infections through immune response; 3) others injectable piezoelectric materials—it can be extended to other kinds of piezoelectric materials, especially injectable nanoparticles, microspheres, and hydrogels. Therefore, our findings provide guidance in immunotherapy and show great potential in clinical translation.

## 4 Experimental Section

##### Materials

A *β*‐PVDF film with a thickness of 52 µm and a *α*‐PVDF film with a thickness of 50 µm, each with a layer of ≈10 nm Au on the surface, were purchased from Zhimk Technology Co., Ltd. (Shenzhen, China).

##### Cell Culture

Human leukemia monocytic (THP‐1) cells, human cervical carcinoma (HeLa) cells, and human hepatocellular carcinoma (HepG2) cells were purchased from the Cell Bank of the Chinese Academy of Sciences (Shanghai, China). Specifically, THP‐1 cells were cultured in 1640 medium containing 10% FBS, 1% penicillin/streptomycin, and 50 × 10^−9^ m *β*‐mercaptoethanol, while HeLa and HepG2 cells were cultured in DMEM containing 10% FBS and 1% penicillin/streptomycin. The cells were maintained at 37 °C in 5% CO_2_ in a saturated humidified atmosphere. According to previous studies, ^[^ ^45^, ^46^ ^]^ PMA was used to prime THP‐1 cells. Briefly, 100 × 10^−9^ m PMA was added to the culture medium for 24 h and cells were gently washed with PBS for three times to remove the extra PMA. Then, fresh cell culture medium was added for next experiments.

##### PFM

The piezoelectric responses of the *β*‐PVDF and *α*‐PVDF films were detected by Bruker Dimension Icon Scanning Probe Microscope with an SCM‐PIT and a Pt‐coated conductive tip in contact mode.

##### Constant d_33_

The constants d_33_ of *α*‐PVDF and *β*‐PVDF were detected by a YE2730A d33 METER and calculated based on the average of ten sites in two samples.

##### Live/Dead Cell Staining

After THP‐1 cells were cultured on TCP, *α*‐PVDF or *β*‐PVDF for 48 h in 24‐well microplates at an initial density of 10^4^ cells per well, the culture medium was changed to serum‐free 1640 medium containing 0.5 × 10^−6^ m calcein AM and 3 × 10^−6^ m propidium iodide. After incubation at 37 °C for 20 min, the cells were washed with PBS and observed under an Olympus IX73 inverted fluorescence microscope.

##### CCK‐8 Assay

After THP‐1 cells were cultured on TCP or *β*‐PVDF for 48 h in 96‐well microplates at a density of 5 × 10^3^ cells per well, the culture medium was replaced with 100 µL serum‐free 1640 medium containing 10% CCK‐8 solution (*n* = 3 per group). After incubation at 37 °C for 1 h, the optical density value was detected at a wavelength of 450 nm by a CMax Plus microplate reader.

##### F‐Actin and Nuclear Staining

After washing with PBS, THP‐1 cells cultured on TCP, *α*‐PVDF or *β*‐PVDF were fixed by 4% paraformaldehyde solution for 15 min and washed with PBS. Then, after permeabilization with 0.1% Triton X‐100 for 5 min and blocking with 5% bovine serum albumin (BSA) solution for 30 min, F‐actin and nuclear staining were performed as previously described.^[^ ^47^ ^]^ Staining images were captured with a ZEISS 800 laser scanning confocal microscope.

##### Calculation of Cell Aspect Ratio

Cell aspect ratio were defined as the ratio of major axis to minor axis. The major axis of cells referred to the longest length of an individual cell, while the minor axis represented for the shortest length. The length of major axis and minor axis was measured by Nano Measurer 1.2 software.

##### THP‐1 Cell Sample Preparation for SEM

After incubation on TCP, *α*‐PVDF or *β*‐PVDF for 48 h in 24‐well plates, THP‐1 cells were prepared for SEM as previously described.^[^ ^47^ ^]^ Finally, the samples were observed under a HITACHI S‐4800 scanning electron microscope.

##### Ultrasound Stimulation Treatment

The ultrasound stimulation parameters were set at 90 W, 80 kHz, and 10 min, and stimulation was produced by an ultrasonic generator. Materials were placed into cell culture plates, then cell culture plates were floated in the water bath which connected with ultrasonic generator. For enhancing M1 polarization experiments, ultrasound was applied once a day for 3 days. For RNA sequencing, ultrasound was applied only once, and cells were lysed after 8 h.

##### RT‐qPCR

After THP‐1 cells were cultured on TCP, *α*‐PVDF or *β*‐PVDF for 3 d and then total RNA was extracted from the THP‐1 cells (2 × 10^5^ cells). RT‐qPCR analysis of one housekeeping gene, *β*‐ACTIN, and five targeting genes, TNF‐*α*, IL‐1*β*, MCP‐1, CCL17, and PIEZO1, was performed using the Light Cycler 96 system. The relative expression levels of the target gene were normalized to the level of the reference gene by the 2^−−ΔΔCt^ method and are expressed as the mean ± SD The primer sequences used for RT‐qPCR are listed in Table S1, Supporting Information.

##### Immunofluorescence Staining

After THP‐1 cells were cultured on TCP or *β*‐PVDF for 3 days, they were washed with PBS, and immunofluorescence staining was performed as previously described.^[^ ^49^ ^]^ iNOS (rabbit monoclonal, Cell Signaling Technology), CoraLite594‐conjugated Arg‐1 (mouse monoclonal, Proteintech) or NF‐*κ*B p65 (rabbit monoclonal, Cell Signaling Technology) primary antibodies were used in this study and diluted as recommended.

##### ELISA

The cell culture medium of all groups was collected on day 3, and proinflammatory cytokine (TNF‐*α* and IL‐1*β*) production was assessed by ELISA kits (Invitrogen) according to the manufacturer's protocols.

##### Western Blot

THP‐1 cells were collected with RIPA buffer (Beyotime Institute of Biotechnology, China) and then centrifuged at 14 000 rpm at 4 °C to obtain total protein. Western blot analysis was performed as previously described^[^ ^48^ ^]^ with iNOS (1:1000, Proteintech), NF‐*κ*B p65 (1:1000, Cell Signaling Technology) and phospho‐p65 (Ser536, 1:1000, Cell Signaling Technology) primary antibodies diluted as recommended. GAPDH was used as a reference protein (1:2000, Proteintech).

##### RNA Sequencing

THP‐1 cells (3 × 10^6^) in the TCP, T+US, *β*‐PVDF, and *β*+US groups were treated with TRIzol Reagent (Invitrogen) at 8 h after ultrasound treatment. The experiments were independently performed three times for each group. RNA sequencing was performed by Beijing Genomics Institute (China). A heat map of gene expression levels in the different samples was drawn with pheatmap (v1.0.8). Briefly, differential expression analysis was performed using DESeq2 (v1.4.5) with a *Q* value ≤ 0.05. To gain insight into the change in phenotype, GO and KEGG enrichment analysis of differentially expressed genes was performed with Phyper based on the hypergeometric test. The significance levels of terms and pathways were corrected with Bonferroni's correction based on a rigorous *Q* value threshold (*Q* value ≤ 0.05).

##### Inhibition of Ion Channels

4‐Aminopyridine (S5028, Selleck) can inhibit a wide variety of voltage‐dependent K^+^ channels, and amiloride HCl (S1811, Selleck) is a blocker of T‐type calcium channels. 4‐Aminopyridine (2 × 10^−3^ m) and amiloride HCl (100 × 10^−6^ m) were added to the medium 1 h before ultrasound treatment.

##### Intracellular Ca^2+^ Staining

THP‐1 cells were cultured in confocal dishes (2 × 10^5^ cells) and divided into the TCP, T+US, *β*‐PVDF, and *β*+US groups. The cells were treated with ultrasound, and after 20 min, intracellular Ca^2+^ was detected using a fluorescence probe (Fluo‐4 AM) according to the manufacturer's protocol. Staining images were captured with a ZEISS 800 laser scanning confocal microscope.

##### Intracellular ROS Staining

THP‐1 cells were cultured in confocal dishes (2 × 10^5^ cells) and divided into the TCP, T+US, *β*‐PVDF, and *β*+US groups. The cells were treated with ultrasound, and after 20 min, the ROS fluorescence was detected using 2,7‐dichlorodihydrofluorescein diacetate (DCFH‐DA) according to the manufacturer's protocol. The cells were observed under a ZEISS 800 laser scanning confocal microscope.

##### Macrophage‐Conditioned Medium for Stimulating Tumor Cells

After macrophages in TCP, T+US, *β*‐PVDF, and *β*+US groups were polarized for 3 d, macrophage‐conditioned medium was collected. The mixture of half macrophage‐conditioned medium and half basal medium of tumor cells were added to stimulate HeLa cells and HepG2 cells for 24 and 48 h. The negative control group (CTR) was cultured in basal medium rather than macrophage‐conditioned medium.

##### EdU (5‐ethynyl‐2'‐deoxyuridine) Assay

After being cultured for 3 d, 1.7 × 10^4^ THP‐1 cells from the TCP, T+US, *β*‐PVDF, and *β*+US groups were scraped and seeded in the upper chambers of a 24‐well Transwell system. A total of 2 × 10^3^ tumor cells were seeded in the lower chambers, and monocultured tumor cells were used as the CTR control group. On day 3, the proliferation of tumor cells was evaluated using the BeyoClick EdU‐488 Cell Proliferation Detection Kit (Beyotime, Jiangsu, China) according to the manufacturer's protocol. The percentage was determined across five randomly selected view‐fields of tested samples.

##### Colony Formation Assay

After being cultured for 3 d, 1.7 × 10^4^ THP‐1 cells from the TCP, T+US, *β*‐PVDF, and *β*+US groups were scraped and seeded in the upper chambers of a 24‐well Transwell system. Cell colony formation was assessed by seeding 100 tumor cells per well in the lower chambers, and monocultured tumor cells were used as the CTR control group. On day 10, the tumor cells were fixed with methanol for 10 min and stained with 1% crystal violet for 30 min.

##### Quantification of Images

The survival rate of THP‐1 cells from live/dead staining images and the percentage of EdU^+^ cells were counted manually. The quantification of intensity in immunofluorescence images and western blot analysis were performed by ImageJ software according to the previous method.^[^ ^49^ ^]^

##### Statistical Analysis

All data were presented as the mean ± SD (*n* = 3, exception for Figures 3i and 4h (*n* = 5)) and were normalized to the respective control group, and differences with *p* < 0.05 were considered significant. The differences were analyzed by one‐way ANOVA in GraphPad Software followed by Duncan's multiple range test. Statistical differences were defined as ^*^ *p* < 0.05, ^**^ *p* < 0.01, and ^***^ *p* < 0.001. The in vitro experiments were performed three times independently.

## Conflict of Interest

The authors declare no conflict of interest.

## Supporting information

Supporting Information

## Data Availability

Research data are not shared.
