# Supplementary material for: Wireless Localized Electrical Stimulation Generated by an Ultrasound‐Driven Piezoelectric Discharge Regulates Proinflammatory Macrophage Polarization
Source: Adv Sci (Weinh). 2021 May 3;8(13):2100962. doi: 10.1002/advs.202100962 (PMC8261497; doi:10.1002/advs.202100962)
Supplement: Supplementary file 1 — Supporting Information [file ADVS-8-2100962-s001.pdf]

© 2021 Wiley-VCH GmbH

ADVANCED SCIENCE
Open Access

Supporting Information

for *Adv. Sci.*, DOI: 10.1002/advs.202100962

Wireless Localized Electrical Stimulation Generated by an Ultrasound-driven Piezoelectric Discharge Regulates Proinflammatory Macrophage Polarization

Ying Kong, Feng Liu, Baojin Ma, Jiazhi Duan, Wenhu Yuan, Yuanhua Sang, Lin Han, Shuhua Wang*, Hong Liu**

Supporting Information

Wireless localized electrical stimulation generated by an ultrasound-driven piezoelectric discharge regulates proinflammatory macrophage polarization

Ying Kong[#], Feng Liu[#], Baojin Ma, Jiazhi Duan, Wenhu Yuan, Yuanhua Sang, Lin Han[*], Shuhua Wang[*], Hong Liu[*]

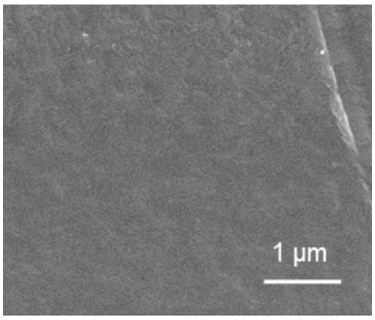

Figure S1. SEM image of α-PVDF.

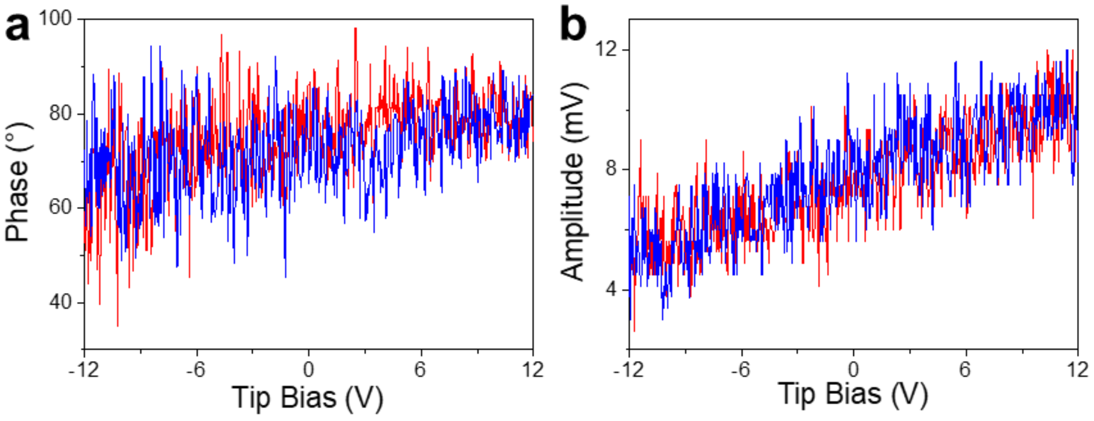

Figure S2. Phase curve (**a**) and amplitude curve (**b**) of α-PVDF at the ramp voltage from −12 V to 12 V.

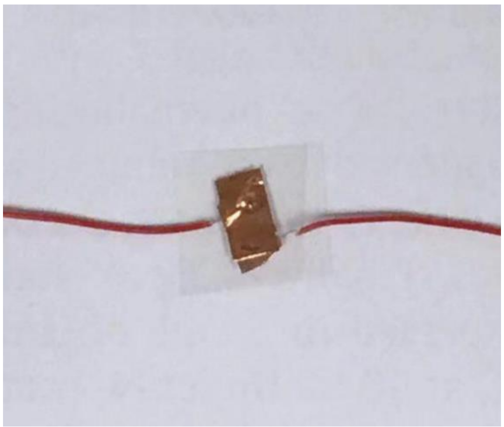

Figure S3. The two sides of β-PVDF were covered with copper foil and connected with wires to detect the output voltage.

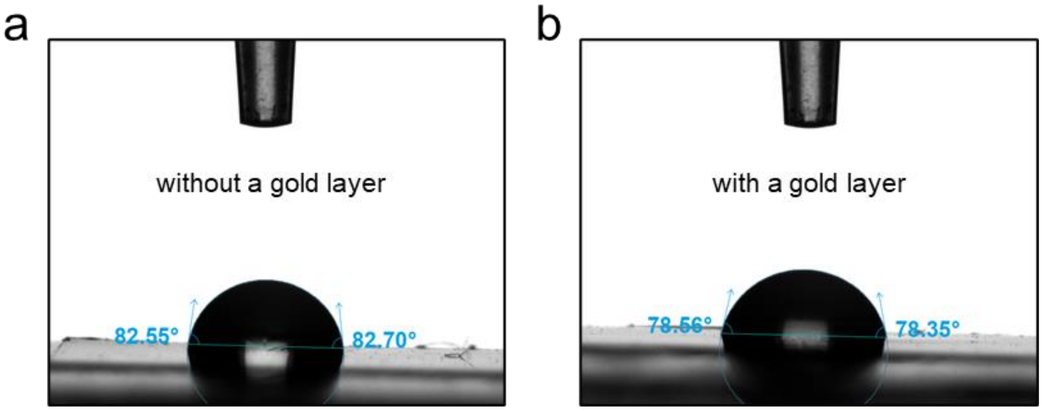

Figure S4. The contact angle of (**a**) β-PVDF film without a gold layer and (**b**) β-PVDF film with a gold layer.

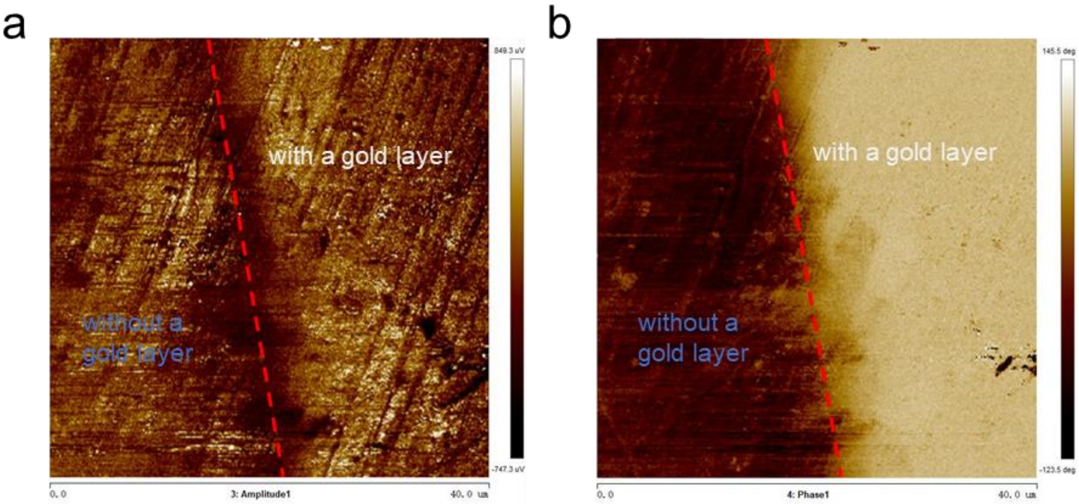

Figure S5. The PFM (**a**) amplitude image and (**b**) phase image of β-PVDF film with and without a gold layer.

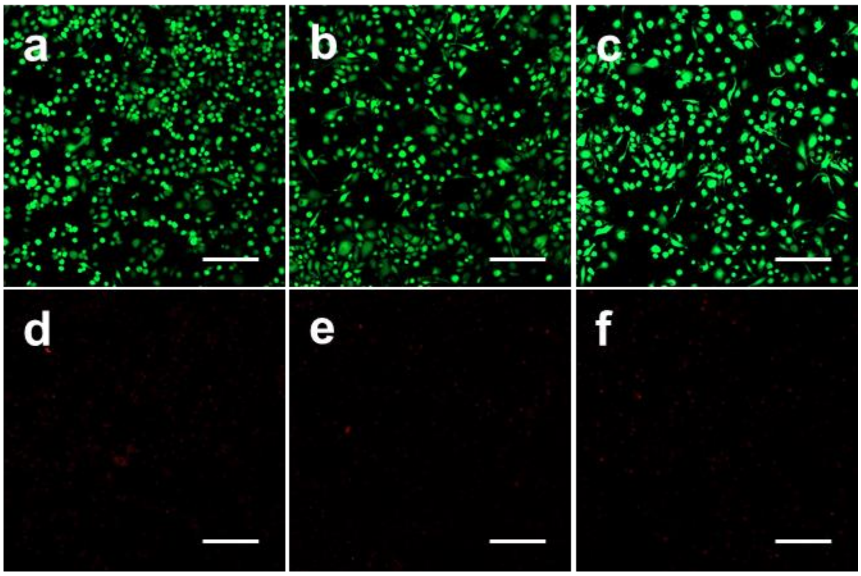

Figure S6. Live (green) cells images of PMA-pretreated THP-1 cells seeded on TCP (**a**), α-PVDF (**b**) and β-PVDF (**c**) and dead (red) cells images on TCP (**d**), α-PVDF (**e**) and β-PVDF (**f**) for 24 h. Scale bar: 200 µm.

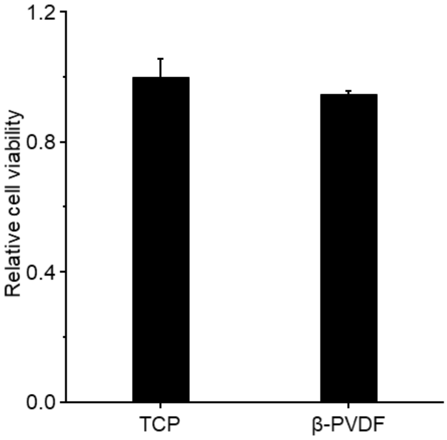

Figure S7. Relatively cell viability of PMA-pretreated THP-1 cells on TCP and β-PVDF for 24 h.

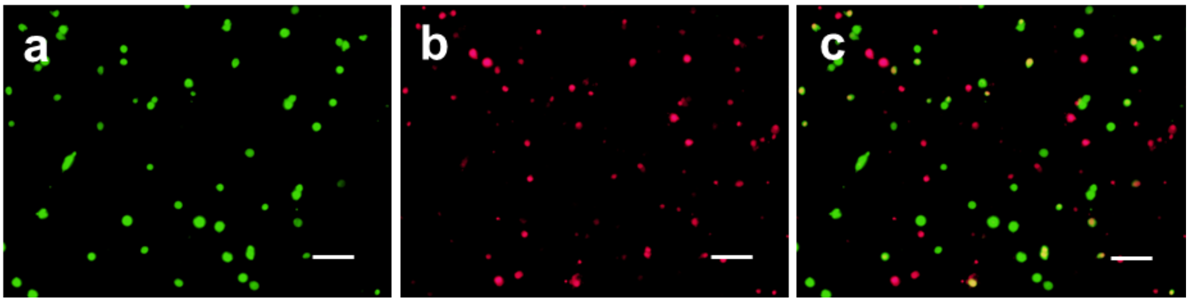

Figure S8. (**a**) Live, (**b**) dead and (**c**) merged staining image for DOX-treated THP-1 cells. Scale bar: 50 μm.

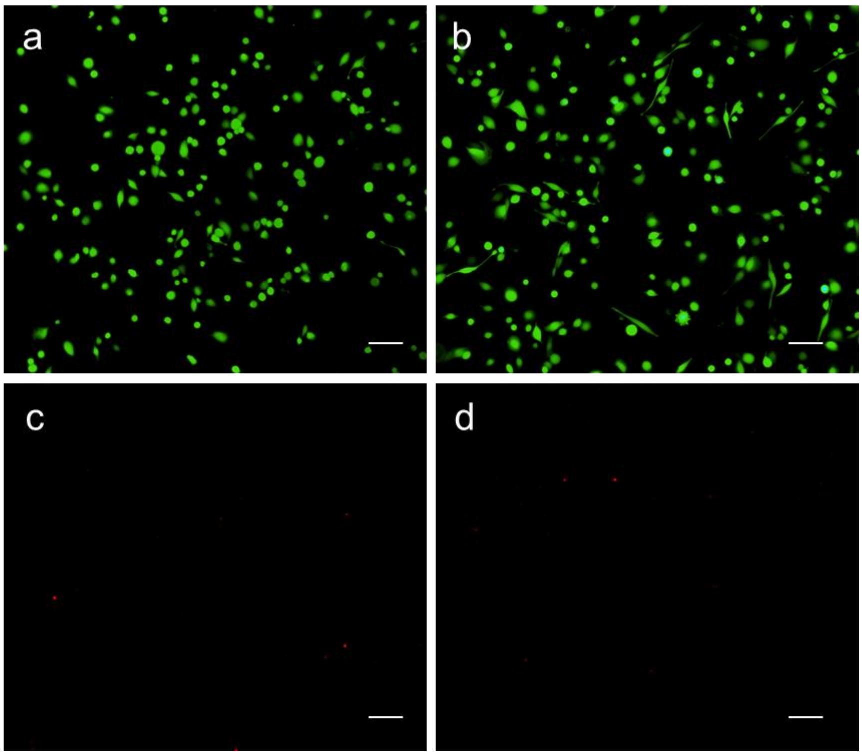

Figure S9. Live (green) cells images of PMA-pretreated THP-1 cells seeded on TCP (**a**), and β-PVDF (**b**) and dead (red) cells images on TCP (**c**), and β-PVDF (**d**) for 72 h. Scale bar: 50 μm.

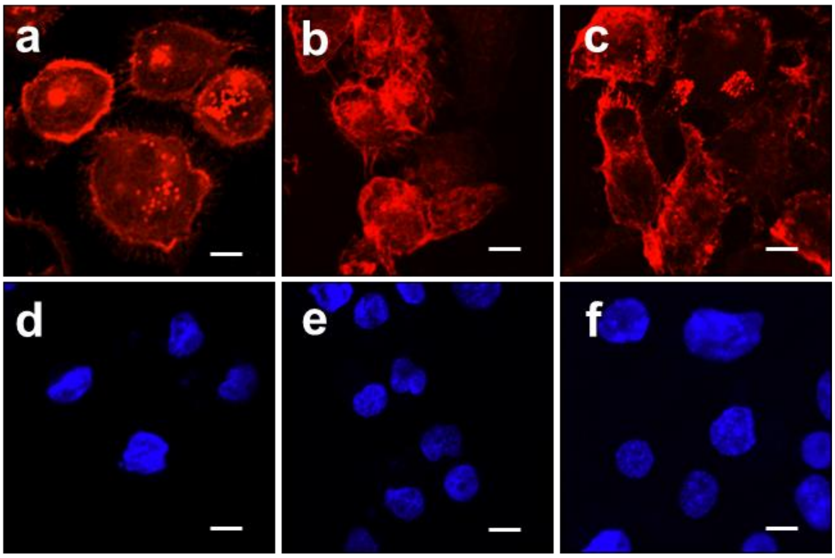

Figure S10. The cellular staining images of F-actin (red) on TCP (**a**), α-PVDF (**b**) and β-PVDF (**c**) and nuclei (blue) on TCP (**d**), α-PVDF (**e**) and β-PVDF (**f**) for 24 h. Scale bar: 10 μm.

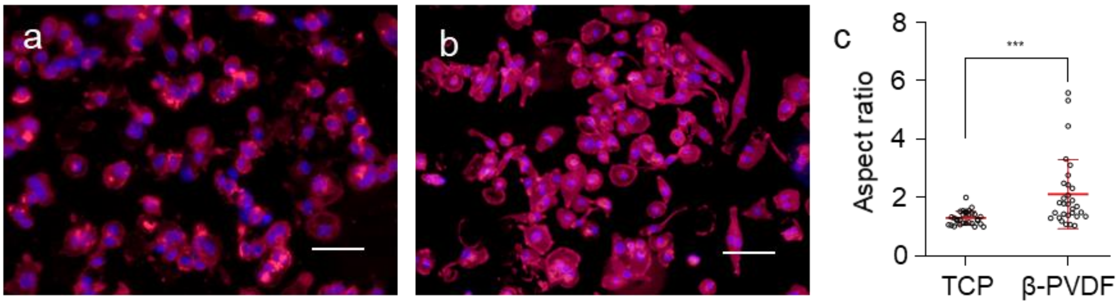

Figure S11. The cellular staining images of F-actin (red) and nuclei (blue) in TCP (**a**) and β-PVDF (**b**) groups for 24 h. Scale bar: 50 μm. (**c**) Qualification of cell aspect ratio on in TCP and β-PVDF groups (***P < 0.001 vs. the TCP control group (n=30)). Three independent experiments were performed and exhibited similar results.

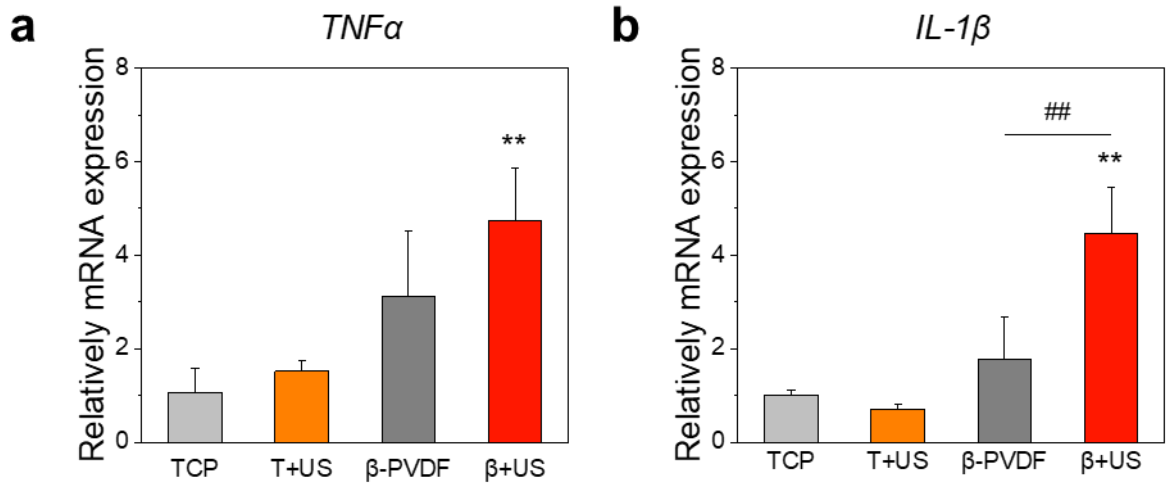

Figure S12. Relative mRNA expression of the M1 markers *TNFα* (**a**) and *IL-1β* (**b**) in macrophages cultured on TCP, T+US, β-PVDF and β+US for 1 d (T, TCP; β, β-PVDF; US, ultrasound stimulation). The data are presented as the mean ± s.d. and were normalized to the level in the TCP group. Significance was determined by unpaired two-tailed Student's t-test (**P < 0.01 vs. the TCP control group, ##P < 0.01 vs. the β-PVDF group (n=3)).

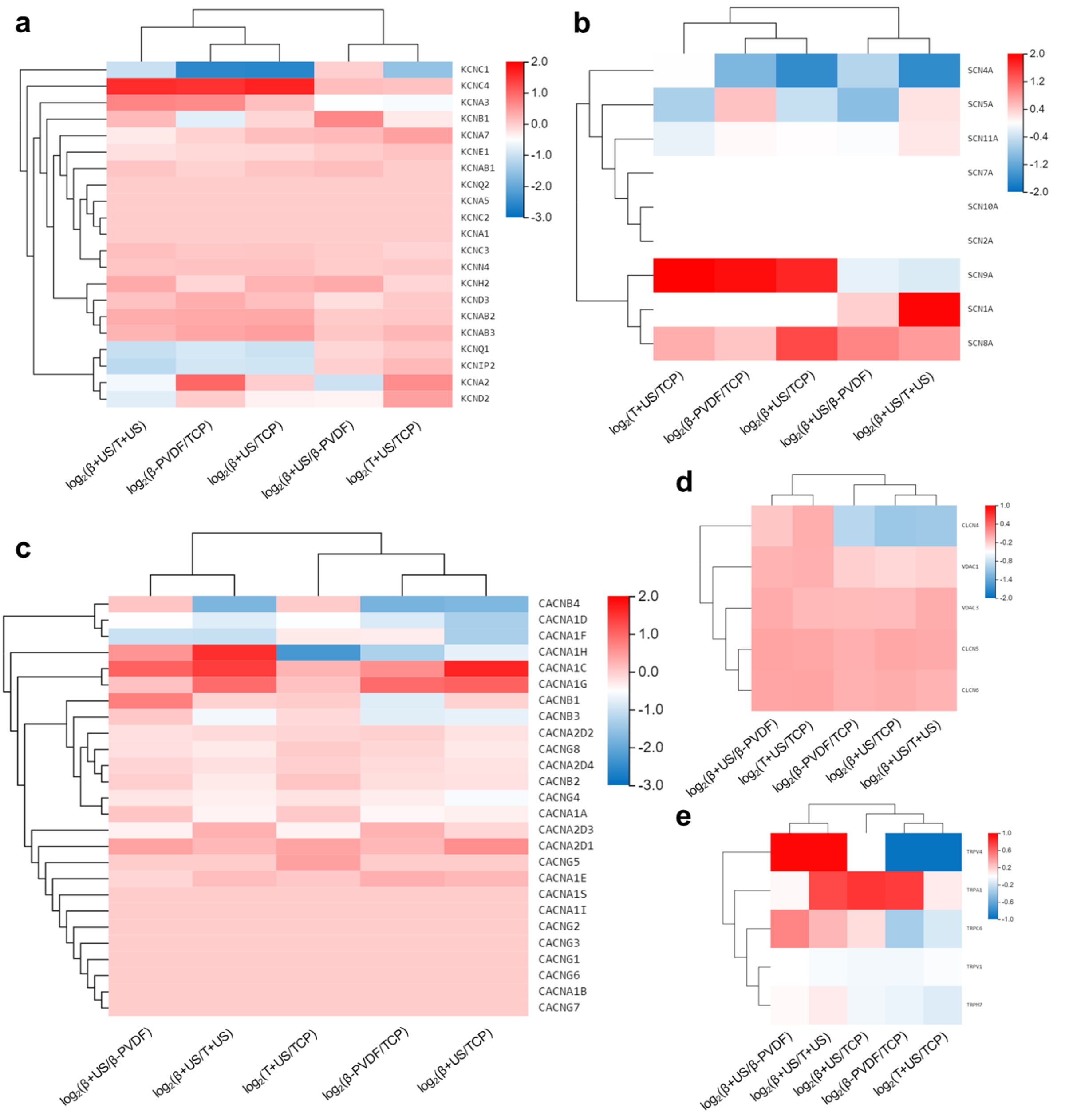

Figure S13. Differential clustering heat maps of (**a**) potassium voltage-gated channels, (**b**) sodium voltage-gated channels, (**c**) calcium voltage-gated channels, (**d**) voltage dependent anion channels and chloride voltage-gated channels, and (**e**) transient receptor potential cation channels.

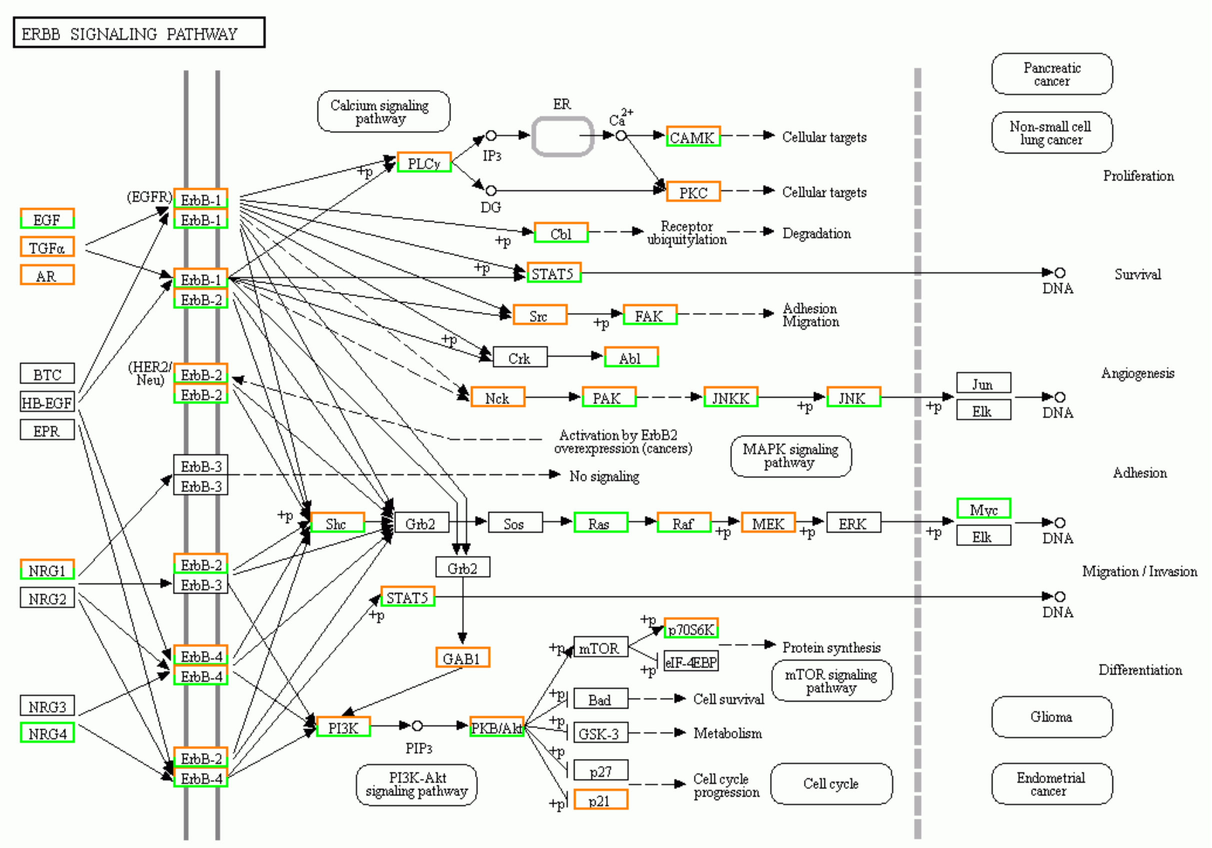

Figure S14. KEGG pathway of enriched ErbB signaling pathway. Red and green outlines represent up-regulated differentially expressed genes (DEGs) and down-regulated DEGs of β+US group compared with TCP group, respectively.

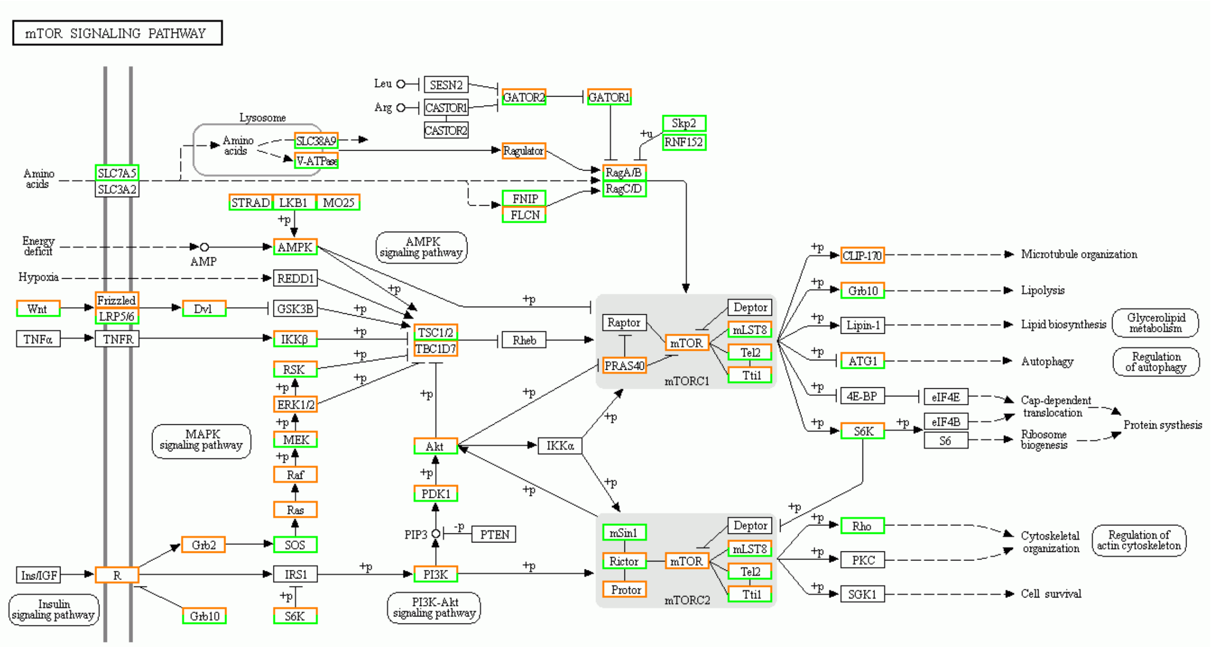

Figure S15. KEGG pathway of enriched mTOR signaling pathway. Red and green outlines represent up-regulated DEGs and down-regulated DEGs of β+US group compared with TCP group, respectively.

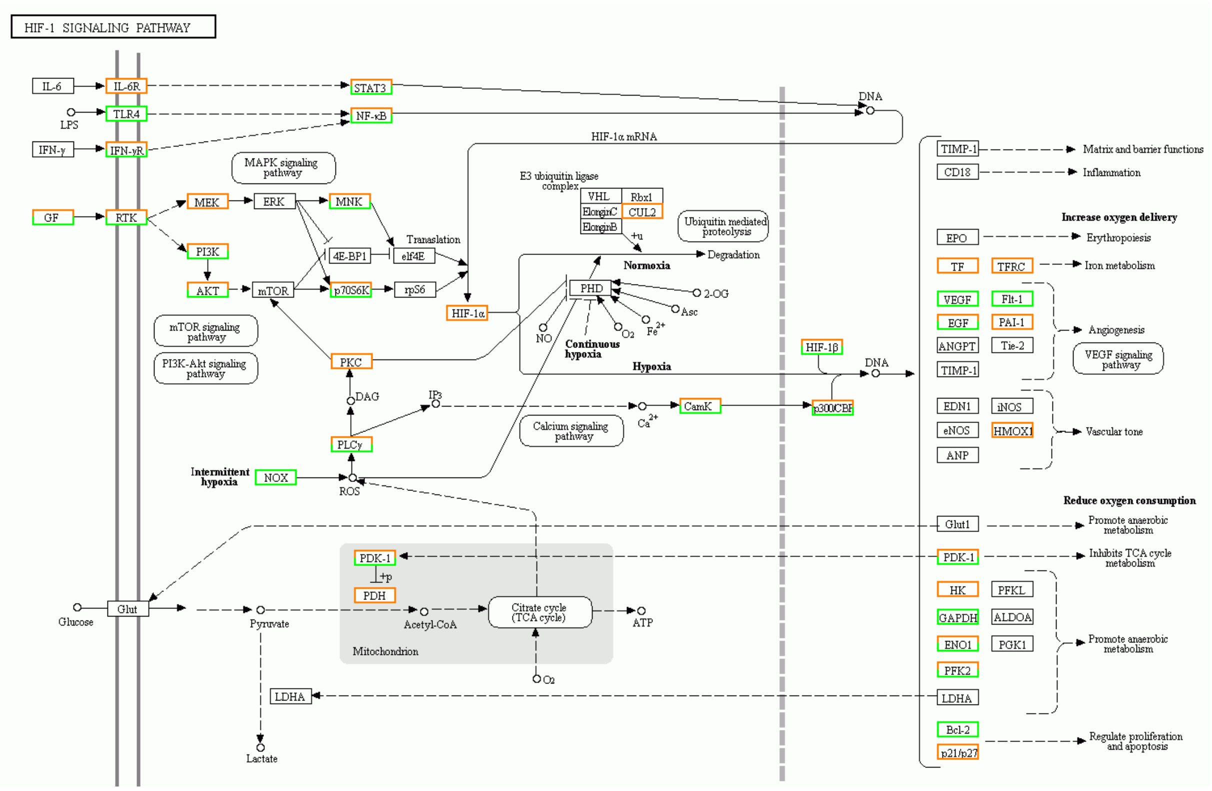

Figure S16. KEGG pathway of enriched HIF-1 signaling pathway. Red and green outlines represent up-regulated DEGs and down-regulated DEGs of β+US group compared with TCP group, respectively.

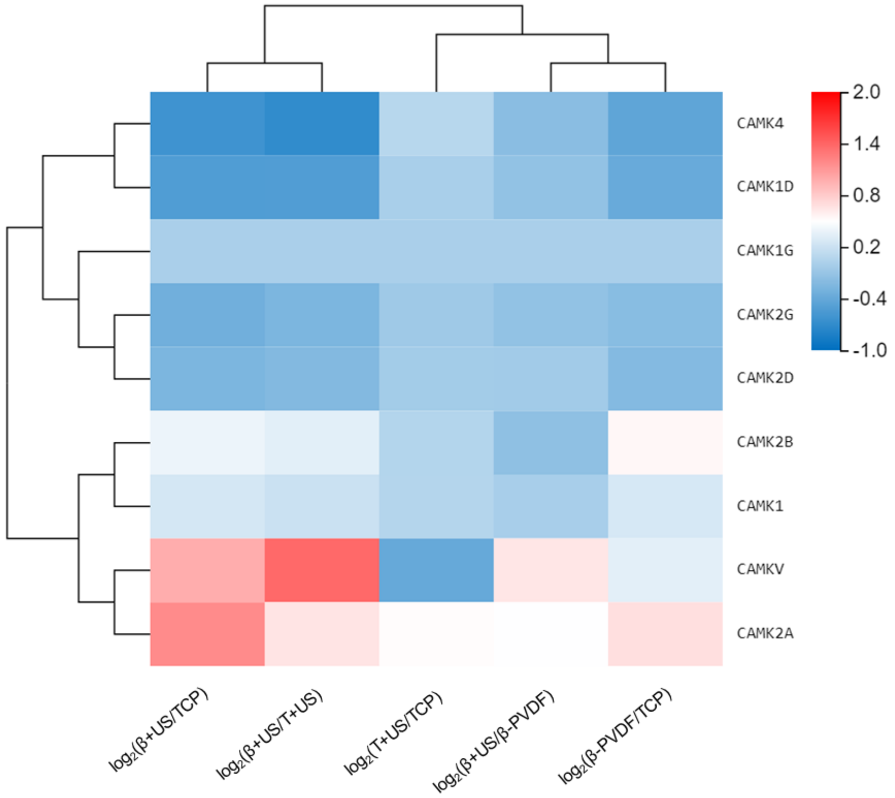

Figure S17. Differential clustering heat maps of calcium/calmodulin kinase family.

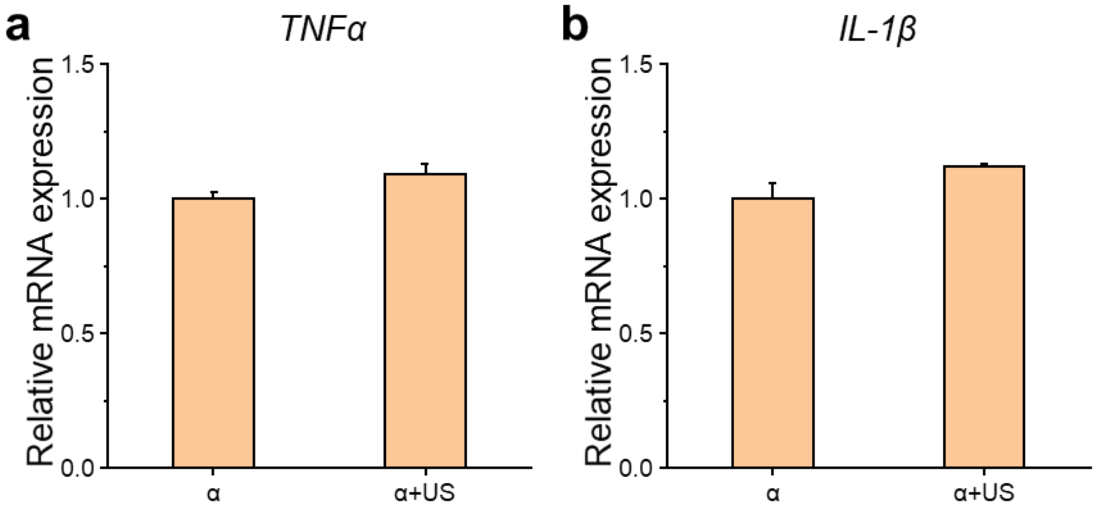

Figure S18. Relative mRNA expression of M1 markers *TNFα* (**a**) and *IL-1β* (**b**) of macrophages cultured on α-PVDF and α+US for 3 d. Data are presented as mean ± s.d. normalized to α-PVDF group.

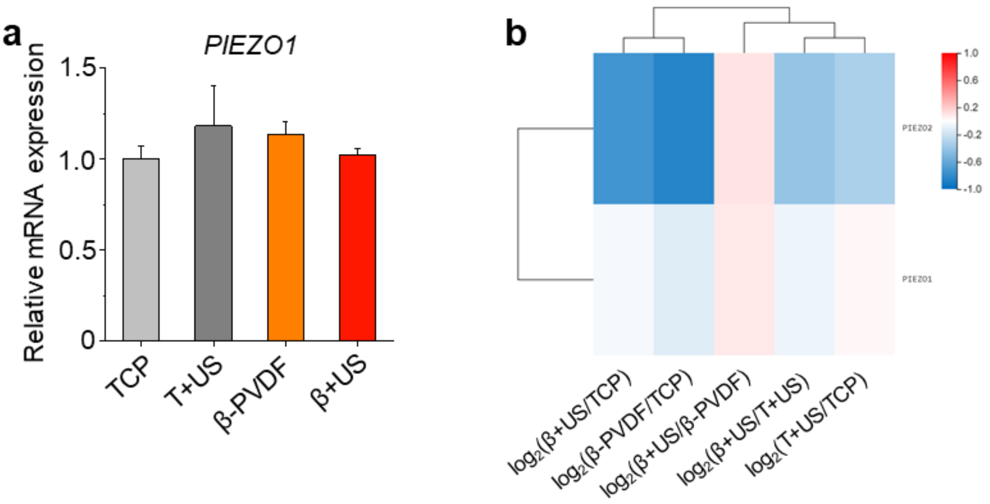

Figure S19. (**a**) Relative mRNA expression of *PIEZO1* of macrophages cultured on TCP, T+US, β-PVDF and β+US for 3 d. (**b**) Differential clustering heat maps of *PIEZO1* and *PIEZO2*.

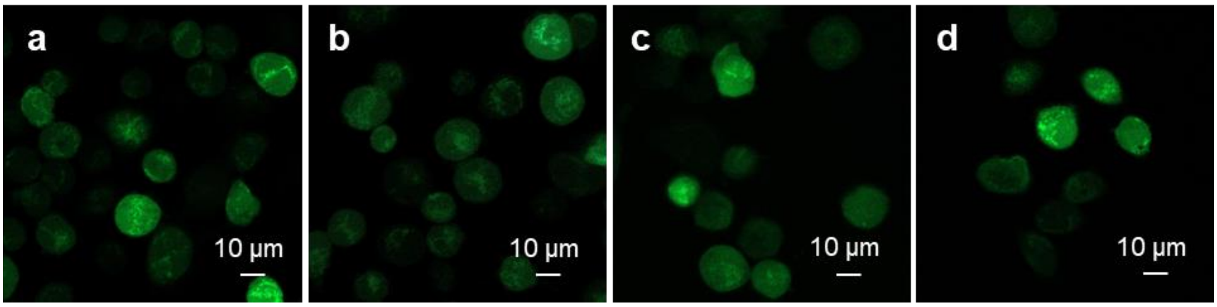

Figure S20. Intracellular ROS level of macrophages in (**a**) TCP, (**b**) T+US, (**c**) β-PVDF and (**d**) β+US groups.

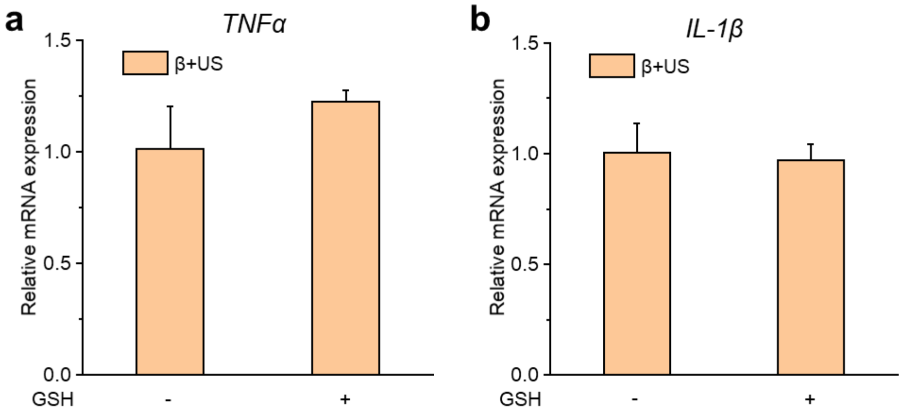

Figure S21. Relative mRNA expression of M1 markers *TNFα* (**a**) and *IL-1β* (**b**) of macrophages cultured on β+US for 3 d with or without GSH for scavenging ROS.

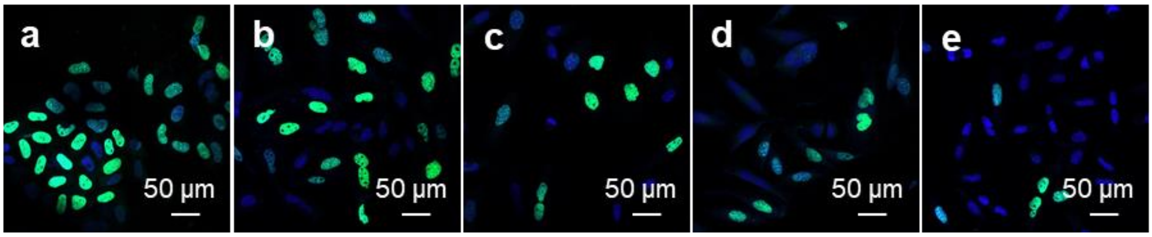

Figure S22. EdU cell proliferation assay of HeLa cells, single cultured (**a**) or co-cultured with macrophages pretreated on TCP (**b**), T+US (**c**), β-PVDF (**d**) and β+US (**e**) for 3 d. EdU has green fluorescence while nuclei blue.

Table S1. The primer sequences used in quantitative RT-qPCR.

Target gene	Primer sequences	
human *β-ACTIN*	Forward	CATGTACGTTGCTATCCAGGC
	Reverse	CTCCTTAATGTCACGCACGAT
human *TNFα*	Forward	CCTCTCTCTAATCAGCCCTCTG
	Reverse	GAGGACCTGGGAGTAGATGAG
human *IL-1β*	Forward	ATGATGGCTTATTACAGTGGCAA
	Reverse	GTCGGAGATTCGTAGCTGGA
human *MCP-1*	Forward	CAGCCAGATGCAATCAATGCC
	Reverse	TGGAATCCTGAACCCACTTCT
human *CCL17*	Forward	CGGGACTACCTGGGACCTC
	Reverse	CCTCACTGTGGCTCTTCTTCG
human *PIEZO1*	Forward	CATCTTGGTGGTCTCCTCTGTCT
	Reverse	CTGGCATCCACATCCCTCTCATC
